# The impact of demographic, clinical, and treatment factors on overall survival in ovarian carcinosarcomas: a National Cancer Database study

**DOI:** 10.3389/fonc.2025.1673861

**Published:** 2025-11-21

**Authors:** Amber Chang, Akaash Surendra, Beau Hsia, Khilen Patel, Joseph Thirumalareddy, Abubakar Tauseef

**Affiliations:** 1 Morsani College of Medicine, University of South Florida, Tampa, FL, United States; 2 School of Life Sciences, Arizona State University, Tempe, AZ, United States; 3 School of Medicine, Creighton University, Phoenix, AZ, United States; 4 Department of Obstetrics & Gynecology, East Carolina University, Greenville, NC, United States; 5 Department of Medicine, Creighton University, Omaha, NE, United States

**Keywords:** ovarian carcinosarcoma (OCS), national cancer database (NCDB), demographics, clinical presentation, treatment, overall survival (OS), gynecologic oncology

## Abstract

**Objective:**

Ovarian carcinosarcoma is a rare and highly aggressive biphasic neoplasm, accounting for less than 1% of ovarian malignancies. This study utilizes the National Cancer Database (NCDB) to examine the impact of demographics, clinical presentation, and treatment on the overall survival of patients diagnosed with ovarian carcinosarcoma.

**Methods:**

A cohort of 420 patients diagnosed with ovarian carcinosarcoma was identified in the NCDB from 2004 to 2020. Patient characteristics and management strategies were analyzed using Kaplan-Meier survival curves, log-rank tests, and multivariable Cox proportional hazard models to determine significance.

**Results:**

Advanced cancer stage was associated with increased mortality risk, as all stage comparisons yielded significant results, ranging from a nearly two-fold increase in risk comparing Stage I and Stage II cancers (HR: 1.96; 95% CI: 1.13 − 3.40; p = 0.017) to over a four-fold increase in Stage I vs. Stage IV hazard ratios (HR: 4.27; 95% CI: 2.64−6.90; p < 0.001). Likewise, increasing Charlson-Deyo comorbidity scores exhibited a trend towards higher mortality, although only the comparison of score 0 to scores ≥ 3 was statistically significant (HR: 6.70; 95% CI: 2.06−21.80; p = 0.002). Furthermore, only select comparisons within different income classes and educational attainment reached statistical significance. Treatment modalities were found to have inconsistent impacts on survival, as primary radiation therapy was associated with unfavorable survival outcomes (HR: 1.54; 95% CI: 1.00−2.37; p = 0.049), while chemotherapy significantly improved outcomes (HR: 0.40; 95% CI: 0.31−0.52; p < 0.001). When analyzed independently, surgical resection was not significantly associated with overall survival (HR: 0.95; 95% CI: 0.51−1.81; p = 0.887).

**Conclusion:**

Tumor staging, comorbidities, and certain treatment modalities were found to be significant predictive factors of ovarian carcinosarcoma survival. Comparisons between age, race, and insurance status were not significantly associated with overall survival.

## 1 Introduction

Ovarian carcinosarcoma, formerly classified as malignant mixed Müllerian tumors (MMMTs), represents a rare and highly aggressive biphasic neoplasm characterized by the coexistence of malignant epithelial and mesenchymal components (1, 2). These tumors account for less than 1% of all ovarian malignancies, with recent epidemiological data showing an incidence increase from 0.029 to 0.073 per 100,000 women between 1999 and 2018 (3).

Histologically, the carcinomatous elements typically manifest as high-grade serous, endometrioid, or undifferentiated carcinomas, while the sarcomatous components may demonstrate homologous (ovarian-derived) or heterologous differentiation, including chondrosarcoma or rhabdomyosarcoma (1, 4). The pathogenesis remains controversial, although molecular evidence supports a monoclonal origin theory, in which both components share *TP53* mutations, suggesting that sarcomatous elements arise through an epithelial-mesenchymal transition (2, 5).

Clinically, these tumors predominantly affect postmenopausal women (median age 62–67 years), although cases occasionally occur in younger patients, particularly those with predisposing factors like prior pelvic irradiation (3, 4). Presenting symptoms typically include abdominal distension, pelvic pain, and rapidly enlarging abdominal masses, with CA-125 levels frequently elevated above 500 U/mL at diagnosis (1, 6). Advanced disease at presentation is common, with 60-70% of patients having stage III/IV disease at initial surgery (3, 7).

Current treatment paradigms emphasize maximal cytoreductive surgery followed by platinum-taxane chemotherapy, mirroring epithelial ovarian cancer protocols (7, 8). The 5-year overall survival remains poor at 42.5%, though this improves to 60.8% for localized disease (3). Prognostic factors include surgical stage, residual tumor burden, and age ≥50 years (3, 4). Emerging data suggest potential benefits from PARP inhibitors in BRCA-mutant cases and anti-angiogenic agents like bevacizumab, though the evidence remains limited to small case series (7, 9).

This study analyzes demographic, clinical, and treatment factors in ovarian carcinosarcoma using National Cancer Database (NCDB) records. Through multivariable analysis of survival outcomes relative to age, tumor stage, treatment modalities, and socioeconomic factors, we aim to identify modifiable predictors of survival in this orphan disease. Our analysis of 420 cases represents the largest prognostic study to date, with the potential to inform risk-adapted therapeutic strategies for this lethal malignancy.

## 2 Materials and methods

This is a retrospective cohort study using patient data within the National Cancer Database (NCDB) for those diagnosed with ovarian carcinosarcoma from 2004 to 2020. The NCDB is a clinical oncology database derived from hospital registry data containing information on patient characteristics, tumor staging, tumor histology, type of first treatment, disease recurrence, and survival from more than 1,500 Commission on Cancer-accredited facilities. This de-identified patient data was made accessible to the authors through the Participant User Data Files program.

Patients with ovarian carcinosarcoma were identified from NCDB data using the ICD-O-3 histology code 8951, which classifies tumors based on their morphological and histological features. Patients were then selected if they had a behavior code of 3 (invasive) and the ICD-10-CM primary site code C569. Patients were excluded from the cohort if they had any concurrent tumors or any missing clinical or demographic data.

### 2.1 Covariates

Patient data were analyzed according to age, sex, race, ethnicity, income, education level, insurance status, tumor size, tumor stage, Charlson-Deyo comorbidity score, primary/adjuvant radiation therapy, and primary/adjuvant chemotherapy. Race was categorized into three groups: White, Black, and Other. The Other race category included American Indian/Alaska Native, Asian (including Chinese, Japanese, Filipino, Hawaiian, Korean, Vietnamese, Kampuchean, Asian Indian/Pakistani, Micronesian, and other/unspecified Asian groups), and Pacific Islander (including Native Hawaiian and other/unspecified Pacific Islander groups). Ethnicity was separately categorized as Hispanic or non-Hispanic, following NCDB classification standards. Income was represented by median household income (2016−2020) based on the patient’s residential zip code at the time of diagnosis. Income was further analyzed in quartiles, with the cutoffs for median household income defined as follows: Q1 (<$46,277), Q2 ($46,277−$57,856), Q3 ($57,857−$74,062), and Q4 (≥74,063). Education level was defined as the percentage of residents within the patient’s residential zip code (2020 data) who did not complete high school and was similarly divided into quartiles: Q1 (≥15.3%), Q2 (9.1%−15.2%), Q3 (5.0%−9.0%), and Q4 (<5.0%). Insurance status was classified into five categories: uninsured, private insurance, Medicare, Medicaid, and other government insurance. Tumor stage (I−IV) was determined using the NCDB analytical stage, which prioritizes the pathologic stage and only uses the clinical stage if pathologic staging is unavailable. The Charlson-Deyo comorbidity score was used to stratify patients into comorbidity groups of 0, 1, 2, and ≥3. For this analysis, primary therapy is the major treatment modality, irrespective of its sequence in combination therapy, while adjuvant therapy is administered after the main treatment with the intent to eradicate remaining cancer cells and prevent recurrence.

The primary outcome was overall survival, defined as the time from diagnosis to death, with censoring at the date of last contact. Independent clinical factors were identified using multivariable Cox proportional hazards regression. Kaplan-Meier curves were generated, and overall survival was estimated at two, five-, and 10-year intervals using life tables. The multivariable Cox model included the following *a priori* variables: age, distance traveled, race, income, education level, insurance status, Charlson-Deyo comorbidity score, NCDB analytical stage, surgical resection of the primary site, primary/adjuvant radiation therapy, and primary/adjuvant chemotherapy. A robust sandwich covariance matrix was employed to account for clustering of patients within facilities. The proportional hazards assumption for each variable was evaluated using log-negative-log survival curves and tests for statistical interaction with time.

### 2.2 Statistical considerations

Cox proportional hazards regression was selected to model survival data due to its ability to handle censored observations and evaluate multiple prognostic factors simultaneously. Kaplan-Meier curves were generated to visualize unadjusted survival differences, while life tables provided standardized survival estimates at clinically actionable timepoints (2, 5, and 10 years). These methods align with recommendations for cancer registry analyses by the NCDB and the American Joint Committee on Cancer (AJCC). The descriptive statistics, unadjusted survival analysis, and multivariable analysis for this study were conducted using IBM Statistical Package for the Social Sciences (SPSS) version 27 (IBM Corp., Armonk, NY). Patients with any missing clinical or demographic factors were excluded from the cohort. Bonferroni correction was used to adjust the p-value threshold for multiple comparisons, ensuring the family-wise error rate remained controlled at α = 0.05. This conservative approach was preferred over false discovery rate control, given the exploratory nature of socioeconomic factor analyses. Multicollinearity among socioeconomic variables (income, education, and insurance status) was assessed using variance inflation factors (VIF) derived from linear regression models. All VIFs were <2.0, well below the threshold of 5.0, indicating no significant collinearity.

### 2.3 Oversight

The University of Arizona Biomedical Institutional Review Board (IRB) reviewed this study (IRB Submission identifier: STUDY00003534, Approval Number: 2001750-01) and determined that it does not involve human subjects research as defined by the Department of Health and Human Services (DHHS) and Food and Drug Administration (FDA) regulations. Consequently, IRB approval and ongoing review were not required.

## 3 Results

The study cohort comprised 420 patients diagnosed with carcinosarcoma of the ovary. Patients were analyzed in terms of demographic and clinical characteristics, as summarized in **Table 1**.

**Table 1:** Clinical and demographic characteristics of 420 patients with ovarian carcinosarcoma.

Variable	Category	N (%)
Age (years)	Mean ± Standard deviation	66.1 ± 11.8
	Median (interquartile range)	66.0 (18)
Sex	Female	420 (100.0%)
Race	White	377 (89.8%)
	Black	27 (6.4%)
	Other	16 (3.8%)
Zip code-level median household income (2016-2020)	< $46,277	53 (12.6%)
	$46,227−$57,856	80 (19.0%)
	$57,857−$74,062	106 (25.2%)
	≥ $74,063	181 (43.1%)
Zip code-level education (% without high-school degree, 2020)	≥ 15.3%	69 (16.4%)
	9.1%–15.2%	116 (27.6%)
	5.0%–9.0%	123 (29.3%)
	< 5.0%	112 (26.7%)
Insurance status	Uninsured	8 (1.9%)
	Private Insurance/Managed Care	186 (44.3%)
	Medicaid	13 (3.1%)
	Medicare	211 (50.2%)
	Other Government	2 (0.5%)
NCDB analytical stage	Stage I	47 (11.2%)
	Stage II	45 (10.7%)
	Stage III	243 (57.9%)
	Stage IV	85 (20.2%)
Charlson-Deyo comorbidity score	0	332 (79.0%)
	1	69 (16.4%)
	2	16 (3.8%)
	≥ 3	3 (0.7%)
Treatment	Received primary chemotherapy	309 (73.6%)
	Received adjuvant chemotherapy	260 (61.9%)
	Received primary radiation	28 (6.7%)
	Received adjuvant radiation	9 (2.1%)
	Received surgical resection	407 (96.9%)
Surgical margins	No residual tumor	162 (38.6%)
	Residual tumor, not otherwise specified	40 (9.5%)
	Microscopic residual tumor	19 (4.5%)
	Macroscopic residual tumor	42 (10.0%)
	Margins not evaluable	63 (15.0%)
	Unknown	81 (19.3%)

*NCDB: National Cancer Database.

*NCDB stage I: small, localized tumor.

*NCDB stage II: larger, localized tumor mass with slight lymph node and tissue involvement. *NCDB stage III: large tumor with regional lymph node and tissue involvement.

*NCDB stage IV: metastatic cancer (cancer spread to distant parts of the body).

The cohort was 100% female. The majority of patients were White (n = 377; 89.8%), followed by Black (n = 27; 6.4%) and Other (n = 16; 3.8%). Most patients (n = 368; 87.6%) identified as non-Spanish or non-Hispanic. The mean age at diagnosis was 66.1 years (95% CI: 65.01−67.27), with a standard deviation of 11.8 years. The majority of patients were distributed within the fourth quartile of median household income (n = 181; 43.1%), followed by 106 (25.2%) patients within the third quartile of income. Regarding education, 69 (16.4%) patients fell within the first quartile of education status, while 112 (26.7%) fell within the fourth quartile. Most treatments (n = 174; 42.0%) were administered at Comprehensive Community Cancer Programs, followed by Academic/Research Programs (n = 122; 29.5%). The majority of patients (n = 274; 65.2%) resided in metropolitan areas with populations exceeding 1 million. In terms of insurance coverage, 186 (44.3%) had private or managed care insurance, followed by Medicare (n = 211; 50.2%) and Medicaid (n = 13; 3.1%).

All patients presented with invasive tumors. Regarding disease stage, 47 (11.2%) patients presented with Stage I disease, and 85 (20.2%) patients presented with Stage IV disease. The cohort exhibited varying comorbidity burdens, with 332 (79.0%) having a Charlson-Deyo comorbidity score of 0. The majority of patients received surgical resection of the primary site (n = 407; 96.9%). The most common surgical procedures include cytoreductive surgery (n = 217; 51.7%), salpingo-oophorectomy with omentectomy (n = 111; 26.4%), and bilateral salpingo-oophorectomy (n = 58; 13.8%). The remainder of patients either did not undergo surgery (n = 13; 3.1%) or received a unilateral salpingo-oophorectomy, pelvic exenteration, or complete tumor resection (n = 21; 5.0%). Postoperative assessment revealed no residual tumor in 162 (38.6%) patients and residual tumor in 101 (24.0%) patients. Residual tumors include macroscopic (n = 42; 10.0%), microscopic (n = 19; 4.5%), and NOS remaining tumor (n = 40; 9.5%). The remaining 37.4% of patients either did not undergo surgery or had margins that were not evaluable.

Of the 314 patients (74.8%) who received surgery and had available surgery sequence data, 209 (66.6%) had upfront surgery, 83 patients (26.4%) only had surgery without further intervention, and 22 (7.0%) patients received surgery after neoadjuvant therapy. The remaining 106 (25.2%) cohort members either did not receive surgery or did not report combination therapy. Radiation therapy was the primary treatment modality for 28 (6.7%) patients, while primary chemotherapy was used for 309 (73.6%) patients. In terms of adjuvant therapies, 9 (2.1%) patients received adjuvant radiation therapy, and 260 (61.9%) received adjuvant chemotherapy.

**Figure 1:**
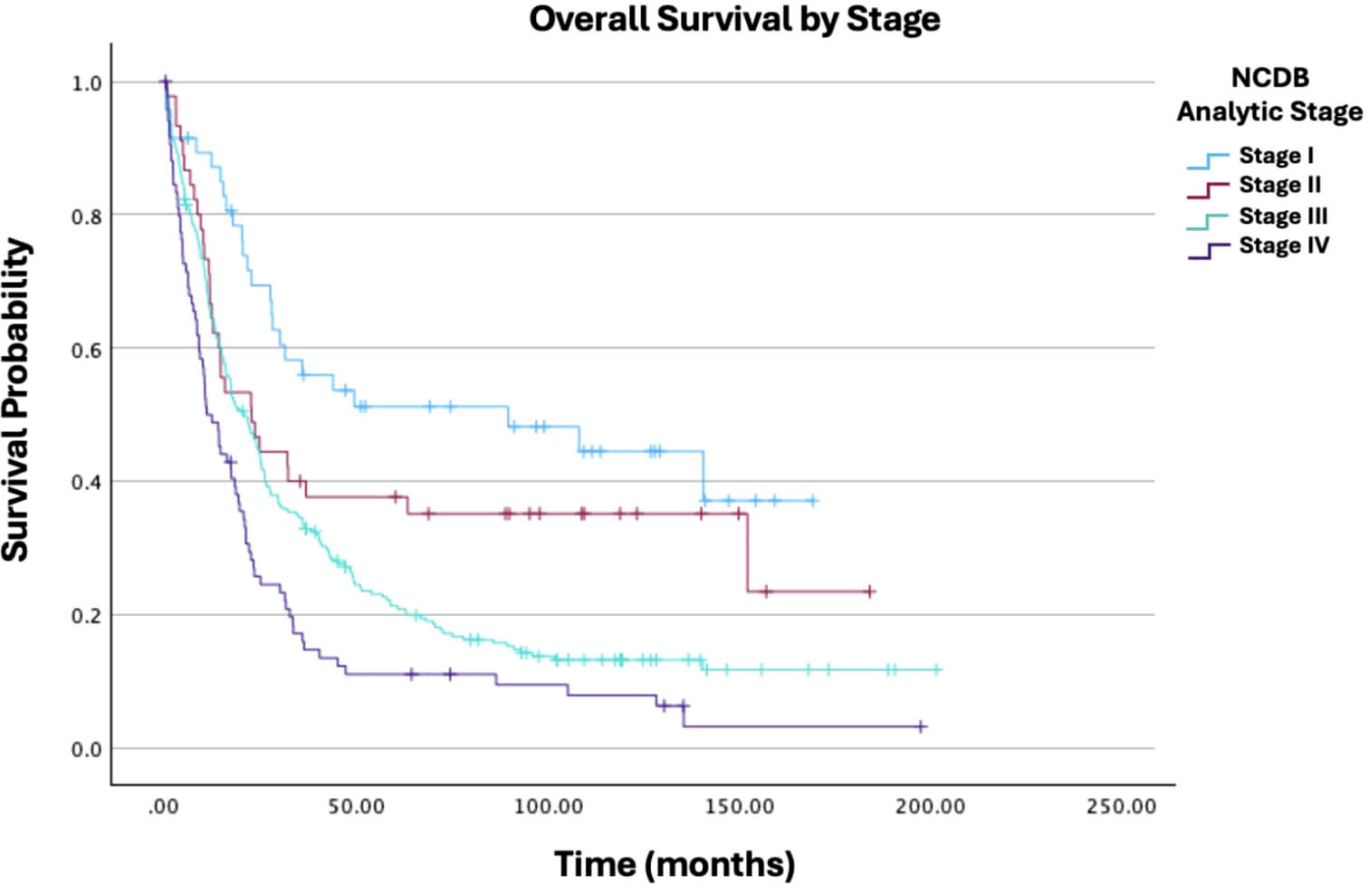
Overall survival by NCDB analytic stage of patients with ovarian carcinosarcoma (N = 420, p < 0.001).

**Figure 2:**
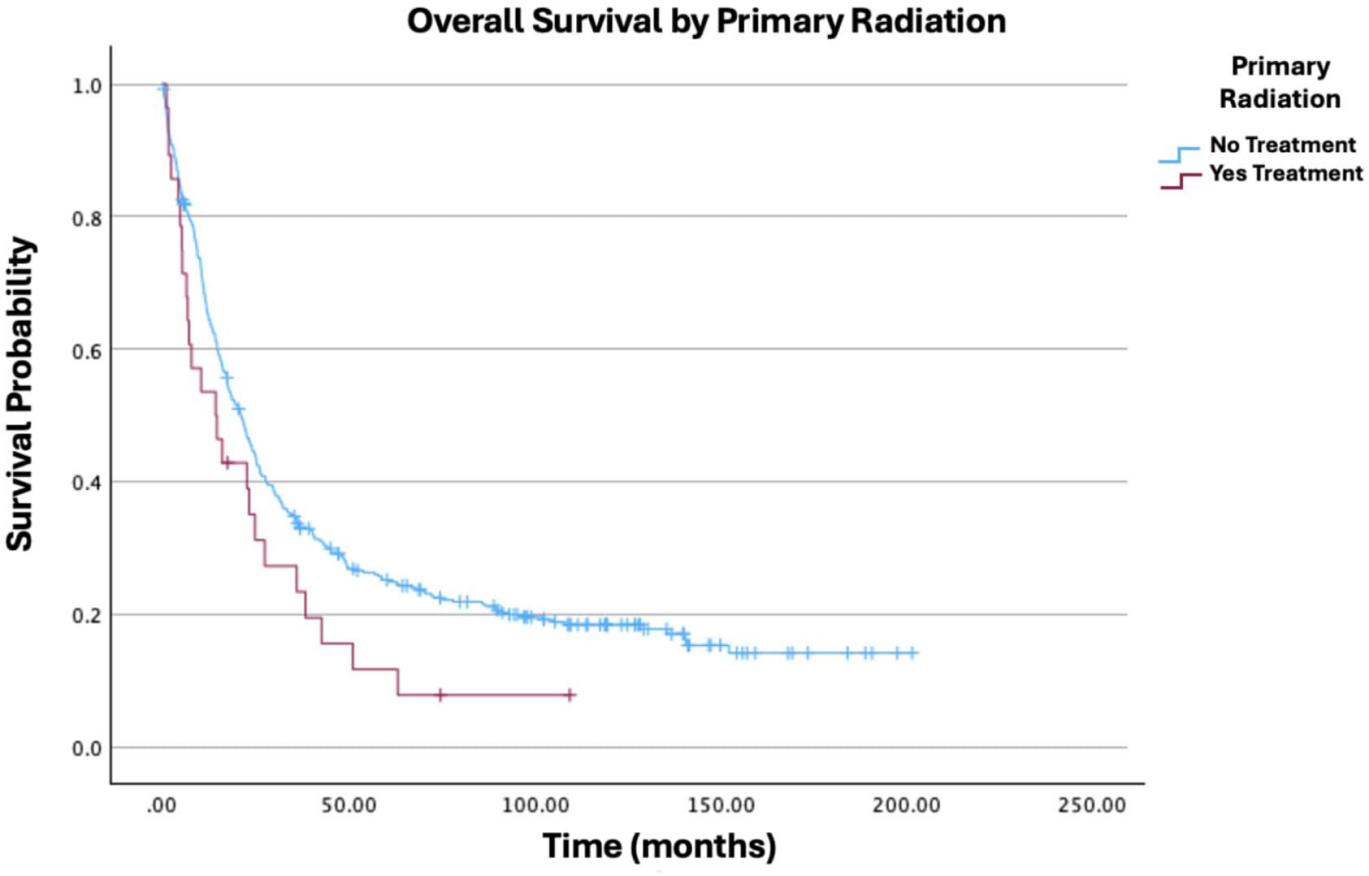
Overall survival by primary radiation of patients with ovarian carcinosarcoma (N = 420, p = 0.049).

**Figure 3:**
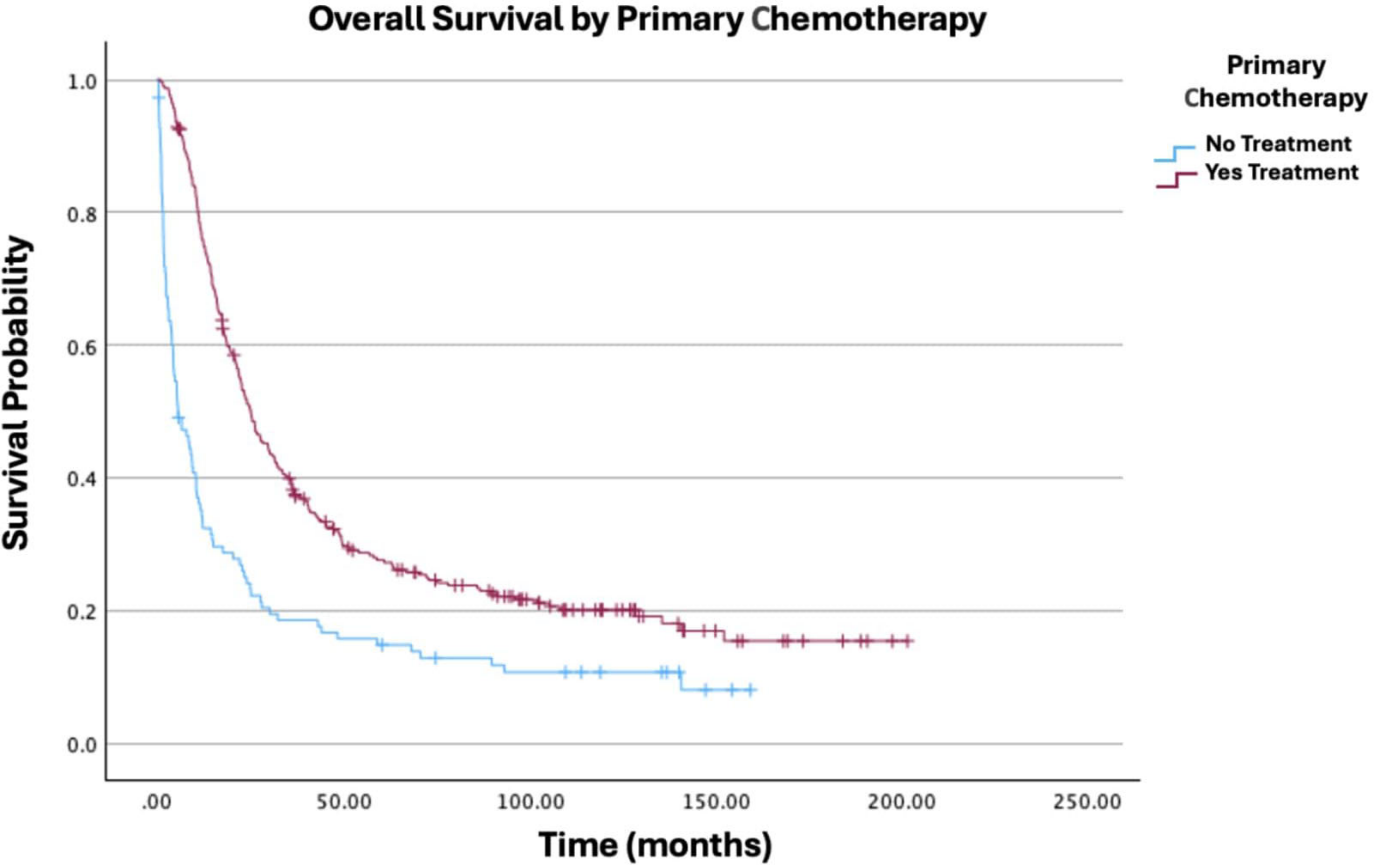
Overall survival by primary chemotherapy of patients with ovarian carcinosarcoma (N = 420, p < 0.001).

**Figure 4:**
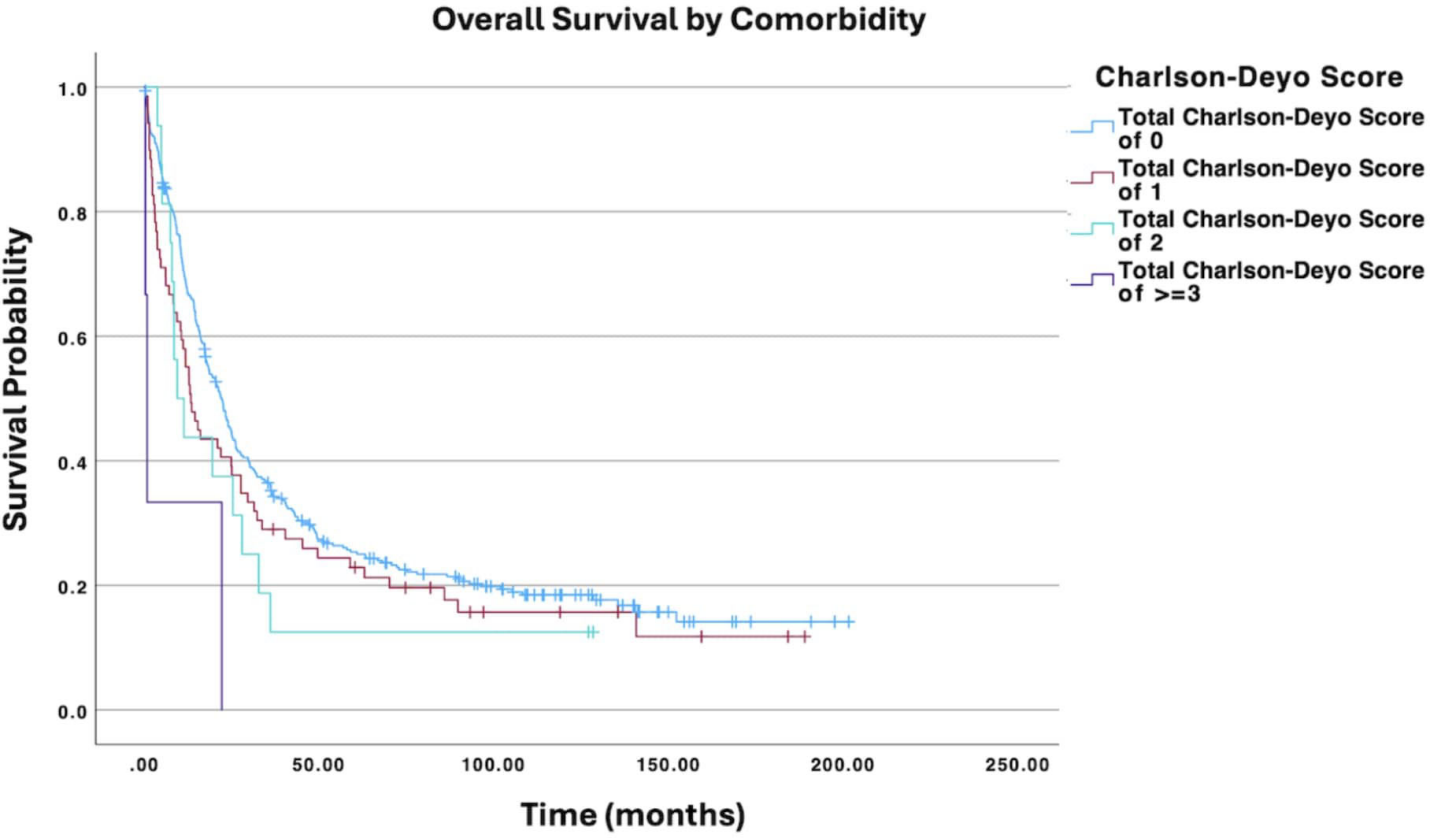
Overall survival by Charlson-Deyo comorbidity scores of patients with ovarian carcinosarcoma (N = 420, p = 0.002).

**Figure 5:**
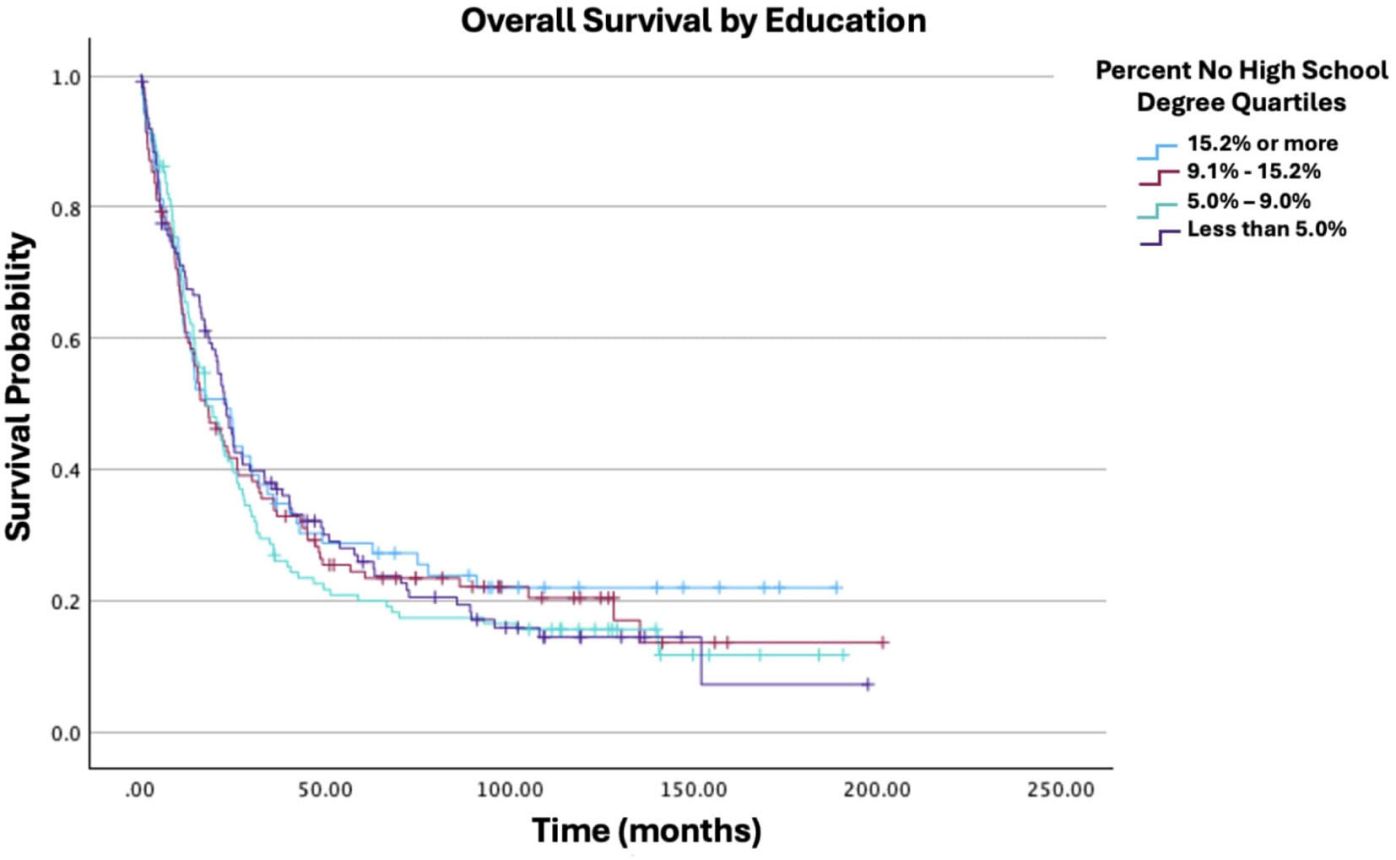
Overall survival by education of patients with ovarian carcinosarcoma (N = 420, p = 0.026).

**Figure 6:**
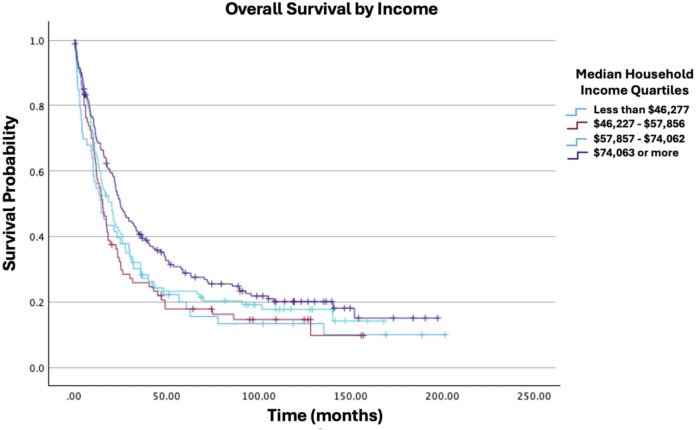
Overall survival by income of patients with ovarian carcinosarcoma (N = 420, p = 0.001).

**Table 2** reveals the multivariate Cox regression model analysis of the variables listed above. The most significant predictors of mortality are displayed in **Figures 1–6**.

**Table 2:** Multivariable Cox regression model of 420 patients with ovarian carcinosarcoma.

Variable	Comparison	HR (95% CI)	p-values
Age (five years)		1.05 (0.99 – 1.10)	0.079
Distance traveled (per 50 miles)		1.048 (0.995 – 1.104)	0.268
Race	White vs. Black	0.77 (0.48 - 1.23)	0.276
	White vs. Other	1.38 (0.80 - 2.39)	0.254
	Black vs. Other	1.79 (0.89 - 3.61)	0.105
Zip code-level median household income (2016-2020)	< $46,277 vs. $46,227−$57,856	0.98 (0.66 - 1.45)	0.905
	< $46,277 vs. $57,857−$74,062	0.69 (0.46 - 1.04)	0.077
	< $46,277 vs. ≥ $74,063	0.51 (0.32 - 0.80)	0.003
	$46,227−$57,856 vs. $57,857−$74,062	0.71 (0.50 - 1.01)	0.059
	$46,227−$57,856 vs. ≥ $74,063	0.52 (0.35 - 0.78)	0.001
	$57,857−$74,062 vs. ≥ $74,063	0.73 (0.52 - 1.02)	0.066
Zip-code level education (% without high-school degree)	≥15.3% vs. 9.1%−15.2%	1.14 (0.80 - 1.64)	0.472
	≥15.3% vs. 5.0%−9.0%	1.57 (1.06 - 2.33)	0.026
	≥15.3% vs. <5.0%	1.62 (1.01 - 2.60)	0.047
	9.1%−15.2% vs. 5.0-9.0%	1.37 (0.98 - 1.93)	0.066
	9.1%−15.2% vs. <5.0%	1.42 (0.93 - 2.15)	0.103
	5.0%−9.0% vs. <5.0%	1.03 (0.73 - 1.45)	0.86
Insurance status	Uninsured vs. Private	1.15 (0.51 - 2.61)	0.731
	Uninsured vs. Medicaid	1.82 (0.67 - 4.96)	0.241
	Uninsured vs. Medicare	0.97 (0.42 - 2.26)	0.947
	Uninsured vs. Other Government	2.17 (0.42 - 11.22)	0.356
	Private vs. Medicaid	1.58 (0.81 - 3.06)	0.177
	Private vs. Medicare	0.84 (0.62 - 1.15)	0.282
	Private vs. Other Government	1.88 (0.44 - 8.00)	0.393
	Medicaid vs. Medicare	0.53 (0.26 - 1.09)	0.086
	Medicaid vs. Other Government	1.19 (0.25 - 5.69)	0.827
	Medicare vs. Other Government	2.23 (0.50 - 9.90)	0.291
NCDB analytical stage	Stage I vs. Stage II	1.96 (1.13 - 3.40)	0.017
	Stage I vs. Stage III	2.80 (1.81 - 4.31)	<0.001
	Stage I vs. Stage IV	4.27 (2.64 - 6.90)	<0.001
	Stage II vs. Stage III	1.43 (0.96 - 2.13)	0.08
	Stage II vs. Stage IV	2.18 (1.39 - 3.41)	<0.001
	Stage III vs. Stage IV	1.53 (1.16 - 2.01)	0.003
Charlson-Deyo comorbidity score	0 vs. 1	1.13 (0.84 - 1.51)	0.428
	0 vs. 2	1.32 (0.74 - 2.37)	0.348
	0 vs. ≥ 3	6.70 (2.06 - 21.80)	0.002
	1 vs. 2	1.17 (0.62 - 2.21)	0.62
	1 vs. ≥ 3	5.95 (1.78 - 19.87)	0.004
	2 vs. ≥ 3	5.07 (1.37 - 18.73)	0.015
Treatment	No chemotherapy vs. chemotherapy	0.40 (0.31 - 0.52)	<0.001
	No radiation vs. radiation	1.54 (1.00 - 2.37)	0.049
	No surgery vs. surgery	0.95 (0.51 - 1.81)	0.887

*HR: Hazard Ratio; CI: Confidence Interval; Statistically significant results (p < 0.05) are highlighted.

*Age: Each 5-year cohort was compared with the 5-year cohort before them, resulting in overall HR.

*Distance traveled: Each 50-mile cohort was compared to the previous 50-mile cohort, resulting in overall HR.

*Chemotherapy: includes both primary and adjuvant chemotherapy.

*Radiation: includes both primary and adjuvant radiation.

*Surgery: includes both primary and adjuvant surgery.

As expected, advanced cancer stage was a highly significant predictor of worse outcomes. Compared to Stage I, all subsequent stages showed increased hazard ratios: Stage II (HR: 1.96; 95% CI: 1.13−3.40; p = 0.017), Stage III (HR: 2.80; 95% CI: 1.81−4.31; p < 0.001), and Stage IV (HR: 4.27; 95% CI: 2.64−6.90; p < 0.001). Other stage comparisons were also significant, as shown in **Figure 1**. In terms of treatment, primary radiation therapy (HR: 1.54; 95% CI: 1.00−2.37; p = 0.049) was associated with worse survival (**Figure 2**). Chemotherapy (HR: 0.40; 95% CI: 0.31−0.52; p < 0.001) was associated with improved survival outcomes (**Figure 3**).

Age at diagnosis was not significantly associated with survival (HR: 1.05; 95% CI: 0.99−1.10; p = 0.079). Similarly, race was not significantly associated with survival in this model (White vs. Black HR: 0.77; 95% CI: 0.48−1.23; p = 0.276). While some trends were observed across insurance statuses, none of the comparisons with the uninsured group reached statistical significance. **Figure 4** displays all Charlson-Deyo comorbidity score comparisons; although increasing comorbidity scores showed a trend towards higher mortality risk, only the comparison of a score of 0 vs. ≥3 was statistically significant (HR: 6.70; 95% CI: 2.06−21.80; p = 0.002); other comparisons (e.g., 0 vs. 1, 0 vs. 2) were not. While some individual comparisons within education level (percentage of residents without a high school degree) and median household income were significant, other comparisons within those categories were not. Specifically, for education, the comparisons of ≥15.3% vs. 5.0%−9.0% (HR: 1.57; 95% CI: 1.06−2.33; p = 0.026) and ≥15.3% vs. <5.0% (HR: 1.62; 95% CI: 1.01−2.60; p = 0.047) were significant, but other comparisons were not (**Figure 5**). For income, the comparisons of <$46,277 vs. ≥$74,063 (HR: 0.51; 95% CI: 0.32−0.80; p = 0.003) and $46,227−$57,856 vs. ≥$74,063(HR: 0.52; 95% CI: 0.35−0.78; p = 0.001) were significant, but other comparisons were not (**Figure 6**). Surgical resection was also not significantly associated with survival (HR: 0.95; 95% CI: 0.51−1.81; p = 0.887).

## 4 Discussion

Ovarian carcinosarcomas are a rare, aggressive subset of ovarian cancer and the second most common primary site of carcinosarcomas in women (10). Existing research on carcinosarcomas is limited due to their low prevalence and frequent misdiagnosis, and ongoing studies prioritize descriptions of patient outcomes through treatment modalities or diagnostic characteristics using histopathology (11). To our knowledge, this is the most comprehensive retrospective study to review demographic, clinical, and treatment factors of ovarian carcinosarcomas.

Our study is consistent with the understanding that ovarian carcinosarcomas are most common in postmenopausal women with a range of 60−80 years (4). From our cohort of 420 individuals from the National Cancer Database (NCDB), we reported a mean age at diagnosis of 66.1 years. While age at diagnosis was not significantly associated with survival in our study, the literature on carcinosarcomas frequently includes conflicting results regarding the role of age in predicting overall survival. Some studies identify age, along with staging and complete tumor excision, as key prognostic factors (12), but age is often compared between patients diagnosed with ovarian carcinosarcoma pre- and post-menopause, which limits firm conclusions about its significance as a prognostic factor (13). Additionally, although age is known to be positively correlated with comorbidities, a review by Minlikeeva et al. found that comorbidities have little impact on ovarian tumors in general due to their poor prognosis (14). Similarly, despite the positive association between Charlson-Deyo comorbidity scores and mortality risk in our study, the only significant difference was observed between patients with no comorbidities and those with a score of ≥3. Notably, the high-risk group was limited to three patients and represents a potential limitation in our analysis. Further study with larger cohorts and better representation across comorbidities is needed to validate this finding.

Despite controversies surrounding age and comorbidities, researchers across all studies have established that stage is a key factor in overall survival. 75% of patients diagnosed with ovarian carcinosarcomas present between International Federation of Gynecology and Obstetrics (FIGO) stages III–IV, as reported in a review by Almond et al. (4), just as 78.1% of patients were diagnosed with stage III−IV tumors in our study. Among our cohort, stages II–IV had significantly increased hazard ratios compared to stage I tumors, demonstrating the significance of stage as a predictor of mortality. Ovarian carcinosarcomas are known to have a worse prognosis than ovarian carcinomas, specifically, as 90% of carcinosarcoma cases above stage I have distant metastases at presentation, and their often-asymptomatic presentation leads to late-stage diagnosis (7, 15). Additionally, in contrast to carcinosarcomas of the uterus, the main site of carcinosarcomas in women, which can be diagnosed via endometrial biopsy prior to surgical resection, ovarian carcinosarcoma is most typically diagnosed following surgery (12).

Surgical resection is the standard treatment for ovarian carcinosarcomas (10, 15), as received by the majority of our cohort (96.9%). However, survival rates are shown to vary greatly depending on the surgery’s success; a comprehensive review of carcinosarcomas by Zheng et al. found an inverse relationship between survival and residual foci (4). Patients with no residual foci following cytoreduction had a median 57-month overall survival, while patients with > 1 cm foci had an 11-month survival rate (4). Overall, 38.6% of patients in our cohort had no residual tumor after receiving complete surgical resection, and our average 5-year survival rate was 24% with a 20.6-month overall survival. It is crucial to note that the overall survival reported in the aforementioned study was contingent on optimal cytoreduction. While most patients in our cohort had no residual tumor, including complete microscopic tumor clearance, 24% of patients had residual tumor, which factors into the overall survival for the entire cohort. Furthermore, the determination that surgical resection was not significantly associated with survival was based on a comparison against the 3.1% of patients who did not receive surgery. Although the necessity of surgery is undisputed, the small size of our non-surgical cohort, as well as the binary categorization of surgical outcome used in this study, limits the overarching conclusions about surgery as an independent factor of survival.

While chemotherapy was significantly associated with improved survival outcomes in this study, the role of chemotherapy for ovarian carcinosarcomas, as well as the optimal combination of chemotherapy, remains unclear (16). As reported in a 2013 study by Lee et al., platinum-based monotherapy and combination chemotherapy with platinum, paclitaxel, and/or ifosfamide are common combinations for ovarian carcinosarcomas, yet only yield a 20% response rate (12). Nevertheless, from the 61.9% of patients in our cohort who received adjuvant chemotherapy, we gathered that chemotherapy was associated with improved survival outcomes. The role of radiation, however, requires further study as well, as 6.7% of our cohort receiving primary radiation were analyzed to have a worse prognosis, and there are no comprehensive studies that have examined the effects of surgery, chemotherapy, and radiotherapy altogether (17). In order for these results to be generalizable to the broader population, further replicative studies with larger sample sizes are warranted.

Similar to other studies of ovarian carcinosarcomas (18), we observed that the dominant racial groups in our study were White (89.9%) and Black (6.4%), both mostly of non-Hispanic ethnicity. While Black patients are more likely to face high rates of comorbidities and have low income, Medicaid, or no insurance, known factors that predict decreased survival in ovarian cancer (19), they only experienced a slight decrease in survival compared to White patients in a study conducted by Rojas et al. (18), and no statistical difference in survival in our study. Additionally, mean household income and educational status did not show significance when analyzed separately. It is crucial to highlight that the majority of patients (42%) in our study were followed at Comprehensive Community Cancer Programs, where treatment for cancers with high observed/expected ratios has higher overall survival compared to centers with a low volume of patients with similar prognosis (20), possibly explaining the lack of significant differences among predicted socioeconomic factors.

Ovarian carcinosarcomas are known for their poor prognosis and low survival rates. Many factors lead to challenges in management, including late diagnosis, advanced staging, and intersectionality. Even known treatment options fail to produce consistent results in patients, as even surgery was only associated with a slight increase in survival, without statistical significance. Further limitations of this study include the retrospective nature of the study, which restricts the generalizability of causal inferences, and potentially incomplete data that limit the scope of our conclusions. Despite how comprehensive the NCDB is, it does not include data from patients diagnosed at non-Commission on Cancer-accredited facilities, also preventing statements about patients diagnosed at such facilities. Regardless, the consistency between our findings and those published in the current literature is reassuring and provides support for our conclusions. Using this review as a framework, future studies can benefit from explorations into the effectiveness of different treatment modalities and socioeconomic status on overall survival for patients diagnosed with ovarian carcinosarcomas.

## 5 Conclusion

This study analyzed various factors associated with overall survival in patients with ovarian carcinosarcoma. Consistent with prior literature, factors correlated with increased mortality include advanced cancer staging, high comorbidity scores, and the administration of primary radiation therapy. Chemotherapy was the only identified prognostic factor to significantly improve survival. Evaluation of other factors, such as age at diagnosis, race, insurance status, and surgical resection, did not reach statistical significance in this study, while comparisons of education status and median household income did not yield consistently significant trends. Future research on ovarian carcinosarcoma could benefit from further evaluation of the aforementioned factors, including combination treatment modalities and socioeconomic status, to corroborate the findings of this study and contribute to a deeper understanding of ovarian carcinosarcoma survival.

## Data Availability

The original contributions presented in the study are included in the article/supplementary material. Further inquiries can be directed to the corresponding author.
